# Insufficient Postoperative Rehabilitation in Patients with Both Proximal Femoral Fracture and Antecedent Mental Illness

**DOI:** 10.31662/jmaj.2019-0055

**Published:** 2020-07-08

**Authors:** Mitsuhiko Takahashi, Joji Iwase, Mitsunobu Abe, Naoko Hashimoto, Hirofumi Kosaka, Hiroshi Egawa

**Affiliations:** 1 Department of Orthopedic Surgery, Tokushima Prefectural Central Hospital, Kuramoto, Japan; 2 Department of Psychiatry, Tokushima Prefectural Central Hospital, Kuramoto, Japan

**Keywords:** proximal femoral fracture, mental illness, rehabilitation, psychiatric ward, Functional Independence Measure

## Abstract

**Introduction:**

Though a combination of proximal femoral fracture and mental illness is likely, the management of this combination is not well established. The aim of this study was to clarify the current disposition of acute care and rehabilitation for patients with this combination of conditions at our institution.

**Methods:**

We retrospectively analyzed the records of 192 patients hospitalized in the psychiatric ward who present with a proximal femoral fracture and an antecedent mental illness. We investigated walking ability prior to injury and after surgery, at discharge from our institution, using the Functional Independence Measure (FIM) score.

**Results:**

Although patients in the psychiatric ward demonstrated postoperative hospital stays approximately 10 days longer than those in the orthopedic ward, more than half of the patients in the psychiatric ward were discharged from our institution with a functional level of complete dependence for walking ability. In addition, nearly 90% of the patients studied were transferred to a psychiatric hospital where no physical therapy or rehabilitation was provided to the inpatients.

**Conclusions:**

At our institution, patients with proximal femoral fracture and antecedent mental illness tended to be discharged with complete dependence in walking ability, often to a psychiatric hospital without physical therapy or rehabilitation. We hope this paper will draw attention to the need for rehabilitation in these patients.

## Introduction

Increasing incidence of proximal femoral fracture, or so-called hip fracture, is a common problem in many countries ^(1), (2)^. Proximal femoral fracture leads to loss of independence and physical function. Thus, in most cases of proximal femoral fracture, to facilitate early weight bearing mobility and independence in activities of daily living, surgical intervention is carried out. Postoperative rehabilitation also results in a significant impact on these outcomes. For proximal femoral fractures, postoperative management including rehabilitation is fairly well established, just as the selection of surgical procedures (e.g. hemiarthroplasty or short proximal femoral nail fixation) for these fractures has been established. Numbers of research studies disclosed the factors that influence the outcome after proximal femoral fracture. Cognitive impairment, which is often seen in the elderly population, is one of the factors impeding the postoperative rehabilitation for the patients with proximal hip fractures ^(3), (4)^. Furthermore, psychotic factor was reported to be associated with poor outcomes after a proximal hip fracture ^(5), (6), (7)^. 

According to a recent report, more than 4 million patients with mental illness are cared for in medical facilities, and approximately 300 thousand are hospitalized in Japan ^(8)^. Mental illness and the psychotropic medications used to treat these conditions were associated with reduced bone mineral density and increased risk of fractures ^(9), (10)^. Consequently, the treatment of proximal femoral fracture in patients with antecedent mental illness is a common occurrence. Although continued specific care and management are required for patients with mental illness, little information is available on the postoperative management of proximal femoral fracture in this population. Furthermore, the medical facilities where fractures can be treated surgically with simultaneous management of mental illness are very limited in the current health insurance system in Japan. The aim of this study was to clarify whether or not postoperative rehabilitation for patients with a proximal femoral fracture and antecedent mental illness was properly carried out in the current disposition at our institution. 

## Materials and Methods

This study was conducted in accordance with the Declaration of Helsinki 1964 and its later amendments. In this retrospective study, we reviewed the records of patients with proximal femoral fracture who were treated surgically at our institution between August 2011 and July 2017 (for 6 years). This study was approved by the institutional ethics committee (Approved code: #18-5 by the Institutional Ethics Committee of Tokushima Prefectural Central Hospital), and comprehensive consent including for publication was obtained from all the patients and the patients’ families. Our institution has a tertiary emergency medical facility and department of psychiatry, in addition to other physical medicine and rehabilitation departments. Our institution is the only such facility in our prefecture and one of the merely 10 institutions which were certified as a psychiatric emergency service unit for the patients with physical complications. All patients in our prefecture with mental illnesses who could not be adequately managed at regular hospitals and/or who presented with proximal femoral fractures requiring surgical management were brought to our institution. 

A total of 878 patients with proximal femoral fractures were treated surgically during the study period. Among them, 192 received treatment for fractures while they were in the psychiatric ward; certified psychiatrist(s) at our institution made the decision to admit them to the psychiatric ward for hospitalization. However, patients with slight to mild clinical features of mental illness that could be managed in the general ward were hospitalized in the orthopedic ward for surgical treatment, where they received psychiatric care through the consultation-liaison psychiatry service. The number of cases of different mental illnesses among the patients in the psychiatric and orthopedic wards was shown in Table 1. Among the patients hospitalized in the orthopedic ward, 124 cases were diagnosed as presenting with slight to mild clinical features of mental illness. Some patients presented with more than one of these disorders. Patient characteristics for the psychiatric ward group are shown in Table 2. Compared with patients hospitalized in the orthopedic ward, patients in the psychiatric ward were significantly younger, more male patients were included, and the majority presented with a femoral neck fracture, which further led to a significantly larger proportion undergoing hemi-replacement arthroplasty and a smaller proportion undergoing osteosynthesis. 

**Table 1.** Case Number of Mental Illness in the Psychiatric and the Orthopedic Wards (If Present).

**Mental illness**	**Psychiatric ward**	**Orthopedic ward**
Schizophrenia	106	5
Dementia	55	105
Intellectual disability or developmental disorder	19	1
Depression or bipolar disorder	19	6
Delirium	0	5
Delusion	0	2
Others	18	0

**Table 2.** Baseline Characteristics of the Patients Hospitalized in the Psychiatric Ward.

Age at the surgery (mean ± SD)	70.0 ± 12.8	
Number of female patients (%)	122	(63.5%)
Number of right side affected (%)	97	(50.2%)
Number of femoral neck fracture (%)	135	(69.9%)
Number of replacement arthroplasty (%)	107	(55.4%)
Number of minimally displaced neck fracture managed with osteosynthesis (%)	31	(23.0%)

Though surgical procedures were performed under general anesthesia, in most cases, in the psychiatric ward group, spinal anesthesia was used in five cases in the psychiatric ward group. No local anesthesia was used in the psychiatric ward group. Each patient received 40-minute-a-day physical therapy on weekdays throughout the hospitalization, according to the clinical pathway for managing proximal femoral fracture established at our institution. In the clinical pathway of our institution, postoperative physical therapy started in principle the day after the surgery in either ward. Early weight-bearing exercise was encouraged and practiced as early as two days postoperatively, regardless of fracture type or surgical procedure, unless the fracture was judged unstable by the surgeon. Since our institution is an acute care hospital, patients who regained stable general condition after operative treatment in the orthopedic ward are considered to be transferred to a convalescent rehabilitation ward outside of our hospital. For patients hospitalized in the psychiatric ward, the criteria for discharge were not well defined, and discharge from our institution was decided according to the capacity of the psychiatric ward permits in the most case. Therefore, the patients were discharged from our institution irrespective of functional recovery in either ward. 

We investigated the walking ability in terms of Functional Independence Measure (FIM) scores ^(11)^ (range, from 1 [totally dependent] to 7 [totally independent]) prior to injury and postoperatively at the time of discharge from our institution. For functional mobility in the FIM, walking and using a wheelchair are listed equivalently, but only walking ability was used in this study. The preinjury FIM score for walking ability was determined based on the medical history provided by the patient, the patient’s family, and/or the staff of the psychiatric hospital where the patient was hospitalized until the onset of the proximal femoral fracture. The FIM score for walking ability at discharge from our institution was assessed by the physical therapist who was in charge of the patient.

Independent predictors consisting of patient age, gender, preinjury FIM score, presence of other complication, operation modality, postoperative hospital stay, and each type of mental illness were evaluated by multi-regression analysis with the FIM score for walking ability at discharge as a dependent variable. After the FIM score at discharge was binarized into independent level (FIM score: 6 and 7) and the other (FIM score: 1 to 5), multiple logistic regression analysis was further applied. In addition, Spearman’s rank correlation coefficient and regression analyses were used to present a linear relationship between the FIM score for walking ability at discharge and the predictors that were introduced as relevant by those multivariate analyses. Unpaired t-test was used for comparison of actual values between the two groups and chi-square test for comparisons of ratios. Statistical analyses were performed using StatView version 5.0 (SAS Institute Inc. Cary NC). A p-value of < 0.05 was considered significant. 

## Results

The length of preoperative waiting time, defined as the number of days from the onset of fracture to the day of surgery, did not differ between the two groups (p > 0.05). However, the length of postoperative hospital stay was approximately 10 days longer for patients hospitalized in the psychiatric ward (Table 3), and this difference was significant (p < 0.001). This meant that patients hospitalized in the psychiatric ward exhibited longer postoperative rehabilitation at our institution. 

**Table 3.** Difference in the Periods of Two Ward Groups.

	Psychiatric ward	Orthopedic ward	P value
Length (days) of preoperative waiting period (mean ± SD)	10.9 ± 7.4	9.5 ± 9.0	.053*
Length (days) of postoperative hospital stay (mean ± SD)	24.4 ± 16.2	14.0 ± 10.7	< .001*

*: unpaired t-test

Of the patients hospitalized in the psychiatric ward, 173 (90.1%) were hospitalized in a private psychiatric hospital at the time of injury. Among the remaining 19 patients, 17 were already diagnosed as presenting with mental illness and were being managed at a psychiatric outpatient clinic under the care of the patient’s family. The distribution of pre-injury FIM scores for walking ability were as follows: 4 patients with FIM 1, 5 with FIM 2, 7 with FIM 3, 17 with FIM 4, 24 with FIM 5, 77 with FIM 6, and 58 with FIM 7. The distribution of FIM scores at discharge was as follows: 66 with FIM 1, 32 with FIM 2, 33 with FIM 3, 27 with FIM 4, 22 with FIM 5, 8 with FIM 6, and 4 with FIM 7. This meant that more than half of the patients were discharged at a functional level of complete dependence (FIM score: 1 or 2) for walking ability, and only six percent reached an independent level (FIM score: 6 or 7).

**Figure 1.**
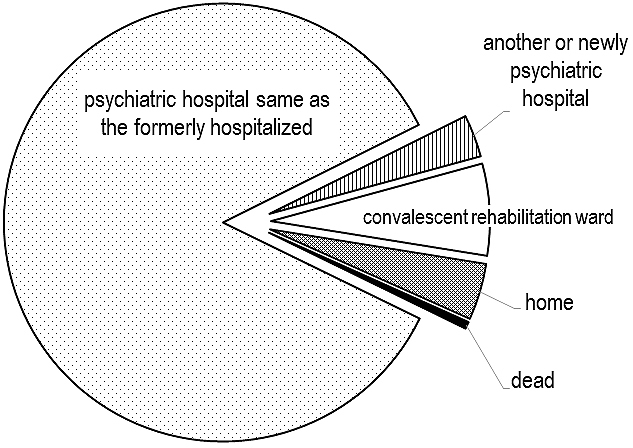
Social and nursing circumstances of patients after discharge from our institution.

After discharge from our institution, 164 patients (85%) were transferred back to the same psychiatric hospital where he or she was at the onset of the fracture (Figure 1). Six patients were transferred to another psychiatric hospital or were newly hospitalized in a psychiatric hospital. Based on our survey at the time of this investigation, no psychiatric hospitals in our prefecture provided inpatients with physical therapy or postoperative rehabilitation. Thirteen of the patients were transferred to the convalescent rehabilitation ward of another hospital for further rehabilitation, because their mental illness was controlled to a point where they no longer required inpatient psychiatric care. Only eight patients were discharged home with the care of their family.

**Figure 2.**
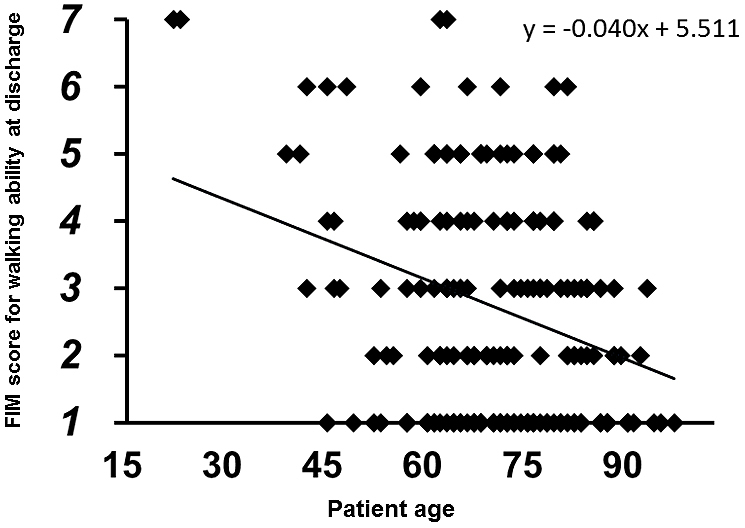
Relationship between FIM score for walking ability at discharge (y-axis) and patient age among the patients hospitalized in the psychiatric ward.

**Figure 3.**
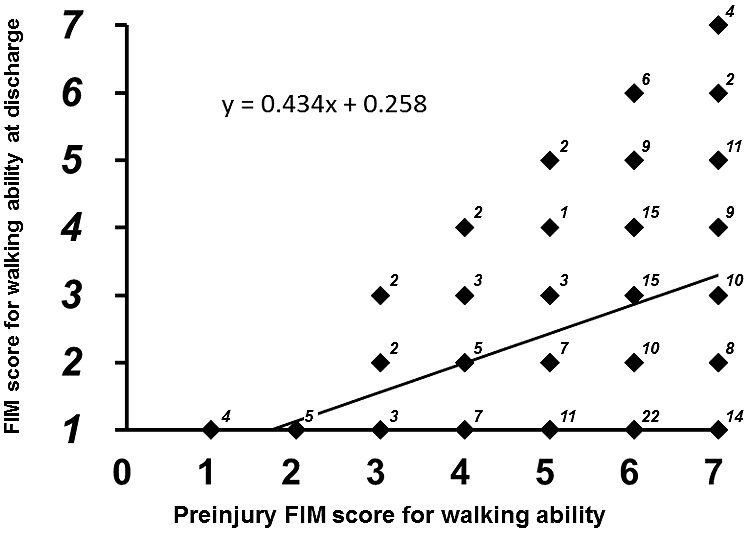
Relationship between FIM score for walking ability at discharge (y-axis) and preinjury FIM score for walking ability among the patients hospitalized in the psychiatric ward. Superscript indicates case number of each dot.

**Figure 4.**
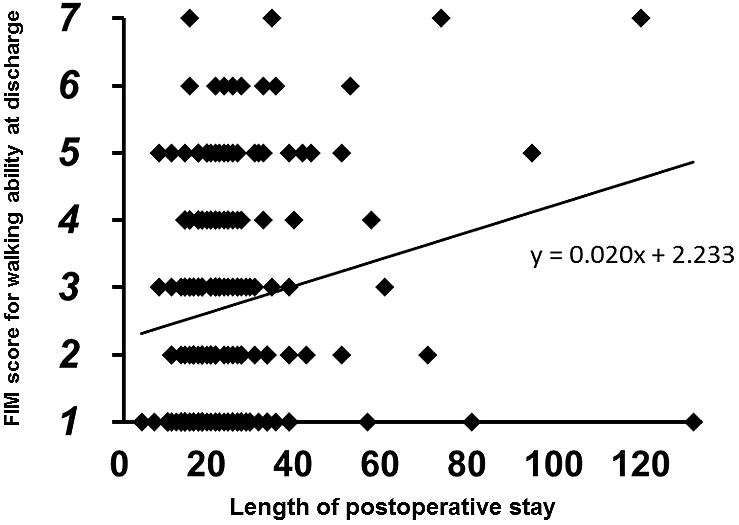
Relationship between FIM score for walking ability at discharge (y-axis) and length of postoperative hospital stay among the patients hospitalized in the psychiatric ward.

No patients showed improvement in FIM scores from preinjury level to the time of discharge. The multi-regression analysis demonstrated that the FIM score for walking ability at discharge was significantly correlated with the independent predictors (r^2^: 0.240, p < 0.0001). Of the independent predictors, patient age (coefficient: –0.044, p = 0.0003) and preinjury FIM score (coefficient: 0.398, p < 0.0001) were indicated as significant predictors. The multiple logistic regression analysis also demonstrated that the binarized FIM score at discharge was significantly correlated with the predictors (r^2^: 0.146, p < 0.0001). In this analysis, postoperative hospital stay (coefficient: 0.002, p = 0.020) was also indicated as a significant predictor in addition to patient age (coefficient: –0.005, p = 0.001) and preinjury FIM score (coefficient: 0.022, p = 0.045). In Spearman’s rank correlation analyses, the FIM score for walking ability at discharge was negatively correlated with patient age (r: –0.207, p = 0.0043, Figure 2) and positively correlated with preinjury FIM score (r: 0.340, p < 0.0001, Figure 3) and postoperative hospital stay (r: 0.195, p = 0.0071, Figure 4).

## Discussion

Proximal femoral fracture, especially in the elderly, is the most common injury in orthopedics. In addition to surgery, rehabilitation exhibits a significant impact on functional recovery ^(12), (13), (14)^. Under the current National Health Insurance of Japan, a proximal hip fracture is one of a limited set of diseases that is eligible for admission to a convalescent rehabilitation ward for postoperative functional recovery ^(15), (16)^. In fact, more than 80% of the patients with proximal femoral fracture hospitalized in the orthopedic ward were transferred to a convalescent rehabilitation ward after discharge from our institution (data not shown), though the length of postoperative hospital stay at our institution was shorter in this orthopedic ward group than in the psychiatric ward group. At the convalescent rehabilitation wards outside of our institution, patients received interdisciplinary and intensive rehabilitation programs to facilitate returning home with sufficient functional recovery. However, this program was hardly applied to patients hospitalized in our psychiatric ward. Eighty-nine percent of these patients were transferred to their original or the other psychiatric hospitals, where no physical therapy or rehabilitation was provided to inpatients because of specialization in psychiatric management as far as we could determine from a survey. Only seven percent of the patients, who were judged no longer required inpatient psychiatric care, were transferred to a convalescent rehabilitation ward for additional rehabilitation in order to gain walking ability. This was a contrasting result even though more than half of the patients were discharged from our institution with functional levels of complete dependence for walking ability (FIM scores 1 or 2), and only six percent of patients reached independent level for walking ability. 

The Ministry of Health, Labour and Welfare of Japan stated that one of the requirements for convalescent rehabilitation wards is that these wards demonstrate a discharge-to-home rate of more than 70% ^(17)^. Most of the patients with proximal hip fracture hospitalized in the psychiatric ward were hospitalized in psychiatric hospitals when the fracture occurred and before admission to our institution. Discharging these patients to their home after a convalescent rehabilitation program would be difficult due to the need for inpatient management of mental illness. Hence, most of them would not be eligible for admission to a convalescent rehabilitation ward. 

Both the length of hospital stay and total accumulated medical charges were reduced in acute care hospitals, since the introduction of the Diagnosis Procedure Combination (DPC)/Per-Diem Payment System under the National Health Insurance system ^(18)^. Additionally, introduction of the convalescent rehabilitation ward probably contributes to these reductions. In contrast, the medical cost for treating a patient with a mental illness is still paid on a fee-for-service basis in the current health insurance system. This study suggested that longer postoperative hospital stay in our hospital correlated with better FIM score at discharge. Thus, as far as the capacity of the psychiatric ward permits, patients in this ward should receive sufficient postoperative rehabilitation stays without concern for the DPC/Per-Diem Payment. Nevertheless, our institution is the only one in our prefecture where both mental illness and severe physical disorders such as hip fracture can be managed by an interdisciplinary team. Therefore, our institution exhibits a huge number of patients with both mental illness and other physical disorders transferred from psychiatric hospitals all over our prefecture. Due to this capacity-related issue in our psychiatric ward, a sufficient period for postoperative rehabilitation was hard to be provided, even though we recognized that the patients gained very limited recovery in walking ability at the discharge from our institution. 

This study presents with several limitations. First, we were unable to perform a long-term follow-up of the patients but simply followed them during the stay in our institution. Although several outpatient follow-up visits occurred for patients who received osteosynthesis for non-displaced to minimally displaced femoral neck fractures, most of the patients were not seen after readmission to the psychiatric hospital. Discharge from our institution at functionally dependent levels may lead to several problems, including the high mortality described in the literature ^(19), (20)^. In contrast, Myers et al. reported that mental illness did not increase the mortality after proximal femoral fracture ^(21)^. Adequate follow-up would be needed to determine whether this is true for the patients, like those in this study. Second, we did not accumulate all the data for other FIM categories, such as other motor and cognitive functions, but only the FIM score for walking ability. Obtaining the preinjury scores in the other FIM categories was often impossible for patients who were transferred from the psychiatric hospital, where the circumstances differ from actual daily living. Third, we did not investigate the details of the patients hospitalized in the orthopedic ward for comparison to those in the psychiatric ward. As described above, most of the patients hospitalized in the orthopedic ward were discharged from our institution to a convalescent rehabilitation ward shortly after surgery, sometimes within a week. Thus, we did not obtain the postoperative FIM score from this group but focused on the psychiatric ward. Finally, this study simply characterized the current disposition of our institution where the preoperative waiting period for either ward was substantially long compared with other countries ^(22)^ and with even Japanese nationwide survey ^(23)^, though our data included the period from the onset of injury to admission. The reason of the delay in surgery at our institution was due to the shortage of available operating rooms and anesthesiologists. The treatment policy for the patients with proximal femoral fractures was reported to differ among hospitals, even within Japan ^(23)^. Moreover, the policy for the patients with a proximal femoral fracture and antecedent mental illness certainly differ. However, to our knowledge, no published reports described the special attributes of postoperative rehabilitation provided for the patients with this combination, even though this combination has been thought to be common and recognized to cause high mortality and poor clinical results ^(5), (6), (7)^. This drove us to write this paper, and we hope this paper will draw attention to this important but neglected matter in the current health insurance system in Japan. Multicenter and nationwide study would be necessary to testify the insufficiency of postoperative rehabilitation for the patients with this combination.

In conclusion, we described the current disposition, at our institution, of patients with proximal femoral fracture in addition to antecedent mental illness. Despite demonstrating a longer postoperative hospital stay than patients with a proximal femoral fracture hospitalized in the orthopedic ward, more than half of patients with this fracture in the psychiatric ward were discharged from our institution with a functional level of complete dependence for walking ability. In addition, nearly 90% of these were transferred to psychiatric hospitals where no physical therapy or rehabilitation was being provided to inpatients. These results were generally predictable in regard to the current health insurance system. Postoperative rehabilitation has been well appreciated for all the patients with proximal femoral. Therefore, patients with proximal femoral fracture should be provided equal opportunities for postoperative rehabilitation regardless of whether or not they present with an antecedent mental illness.

## Article Information

### Conflicts of Interest

None

### Author Contributions

MT contributed to designing the study, analyzing and interpreting of data, and writing the manuscript. JI, MA and HK contributed to data acquisition. NH and HE critically revised the manuscript.

### Approval by Institutional Review Board (IRB)

#18-5 by the Institutional Ethics Committee of Tokushima Prefectural Central Hospital
